# 3,4-Dimeth­oxy-4′-methyl­biphen­yl

**DOI:** 10.1107/S1600536813008957

**Published:** 2013-04-10

**Authors:** Manu Lahtinen, Sami Nummelin

**Affiliations:** a University of Jyväskylä, Department of Chemistry, PO Box 35, FI-40014 JY, Finland; b Molecular Materials, Department of Applied Physics, School of Science, Aalto University, PO Box 15100, FI-00076 Aalto, Finland

## Abstract

In the title compound, C_15_H_16_O_2_, the dihedral angle between the planes of the aromatic rings is 30.5 (2)°. In the crystal, mol­ecules are linked *via* C—H⋯O hydrogen bonds and C—H⋯π inter­actions, forming a two-dimensional network lying parallel to (100).

## Related literature

For structural studies of related biphenyl derivatives, see Lahtinen *et al.* (2013*a* ▶,*b* ▶,*c* ▶); Li *et al.* (2012*a* ▶,*b* ▶). For details of the synthesis, see: Percec *et al.* (2004 ▶, 2006 ▶); Wolfe *et al.* (1999 ▶). For details of various cross-coupling reactions, see: Corbet & Mignani (2006 ▶); Miyaura *et al.* (1981 ▶); Miyaura & Suzuki (1995 ▶); Percec *et al.* (2004 ▶); Wolfe *et al.* (1999 ▶). For self-assembling supra­molecular dendrons based on 3,4-branched bi­phenyls, see: Percec *et al.* (2006 ▶, 2007 ▶). For hollow supra­molecular dendrimers, see Peterca *et al.* (2006 ▶); Percec *et al.* (2008 ▶). For dendritic polyaryl esters, see Nummelin *et al.* (2000 ▶).
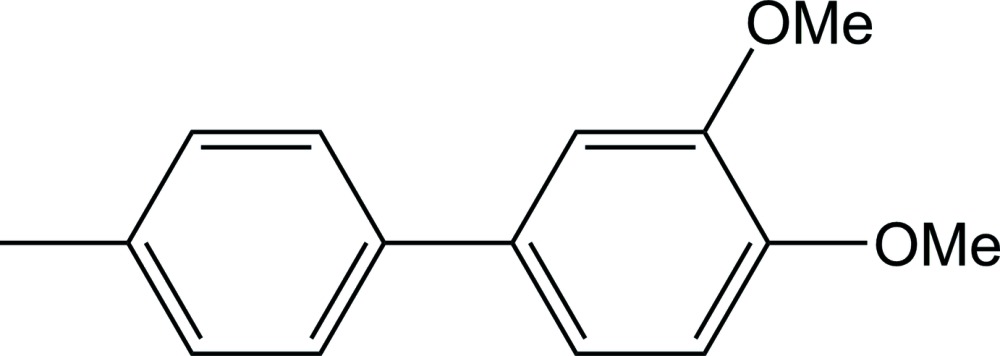


## Experimental

### 

#### Crystal data

C_15_H_16_O_2_
*M* *_r_* = 228.28
Monoclinic, 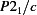

*a* = 17.7430 (9) Å
*b* = 8.7581 (3) Å
*c* = 8.1135 (3) Å
β = 101.795 (5)°
*V* = 1234.17 (9) Å^3^
*Z* = 4
Cu *K*α radiation
μ = 0.64 mm^−1^
*T* = 123 K
0.36 × 0.26 × 0.04 mm

#### Data collection

Agilent SuperNova (Dual, Cu at zero, Atlas) diffractometer
Absorption correction: analytical (*CrysAlis PRO*; Agilent, 2010 ▶) *T*_min_ = 0.880, *T*_max_ = 0.977
4273 measured reflections
2282 independent reflections
1844 reflections with *I* > 2σ(*I*)
*R*_int_ = 0.038

#### Refinement

*R*[*F* ^2^ > 2σ(*F* ^2^)] = 0.052
*wR*(*F* ^2^) = 0.143
*S* = 1.07
2282 reflections
157 parameters
H-atom parameters constrained
Δρ_max_ = 0.23 e Å^−3^
Δρ_min_ = −0.31 e Å^−3^

### 

Data collection: *CrysAlis PRO* (Agilent, 2010 ▶); cell refinement: *CrysAlis PRO*; data reduction: *CrysAlis PRO*; program(s) used to solve structure: *SHELXS97* (Sheldrick, 2008 ▶); program(s) used to refine structure: *SHELXL97* (Sheldrick, 2008 ▶); molecular graphics: *OLEX2* (Dolomanov *et al.*, 2009 ▶) and *Mercury* (Macrae *et al.*, 2006 ▶); software used to prepare material for publication: *OLEX2*.

## Supplementary Material

Crystal structure: contains datablock(s) I, global. DOI: 10.1107/S1600536813008957/fj2625sup1.cif

Structure factors: contains datablock(s) I. DOI: 10.1107/S1600536813008957/fj2625Isup2.hkl

Supplementary material file. DOI: 10.1107/S1600536813008957/fj2625Isup3.cml

Additional supplementary materials:  crystallographic information; 3D view; checkCIF report

## Figures and Tables

**Table 1:** Hydrogen-bond geometry (Å, °) *Cg*2 is the centroid of the C8–C13 ring.

*D*—H⋯*A*	*D*—H	H⋯*A*	*D*⋯*A*	*D*—H⋯*A*
C15—H15*A*⋯O16^i^	0.98	2.57	3.389 (2)	141
C15—H15*C*⋯O16^ii^	0.98	2.42	3.356 (2)	160
C4—H4⋯*Cg*2^ii^	0.95	2.96	3.7807 (18)	145
C17—H17*B*⋯*Cg*2^iii^	0.98	2.83	3.7119 (19)	150

Symmetry codes: (i) 
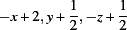
; (ii) 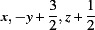; (iii) 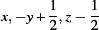.

**Fig. 1.**
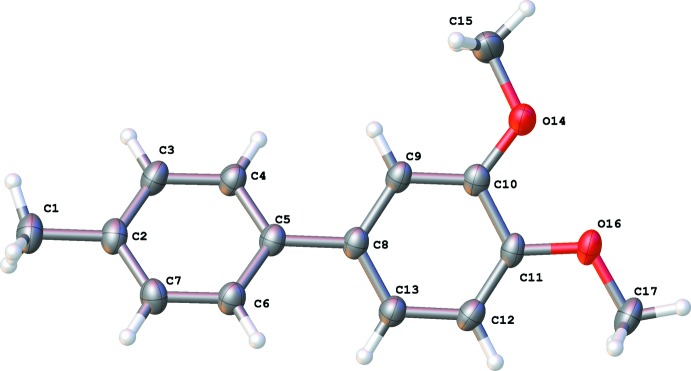
The molecular structure and atomic labeling of the title compound showing 50% probability displacement ellipsoids.

**Fig. 2.**
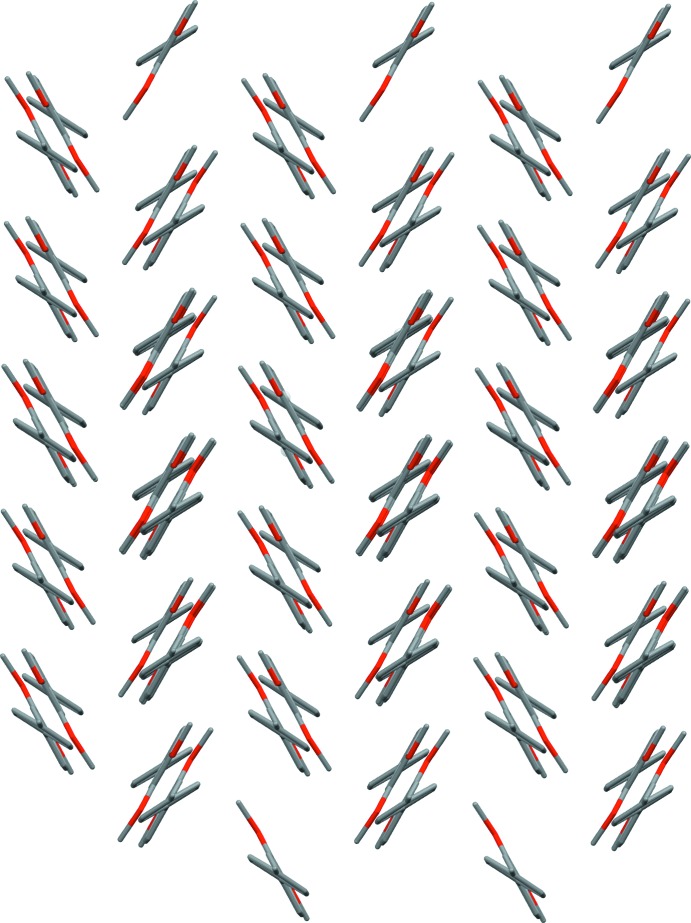
In zigzag formation packed molecule rows along (201)-plane. Hydrogen atoms are omitted for clarity.

**Fig. 3.**
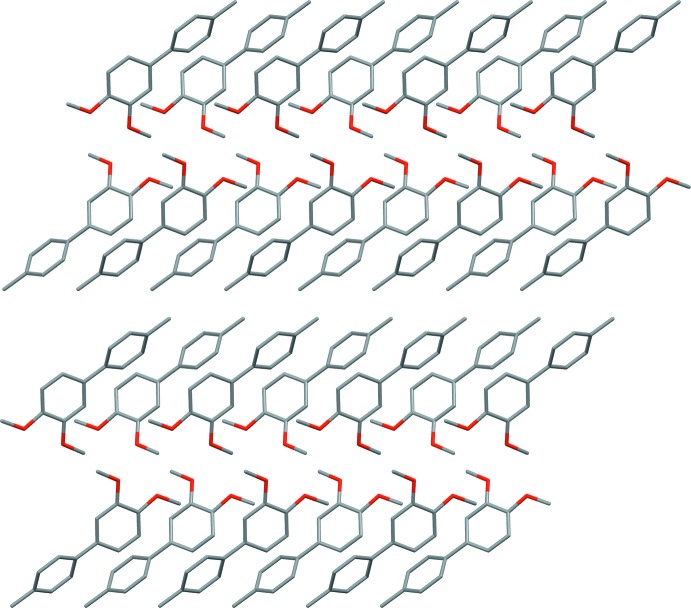
Packing of molecules along b-axis. Hydrogen atoms are omitted for clarity.

**Fig. 4.**
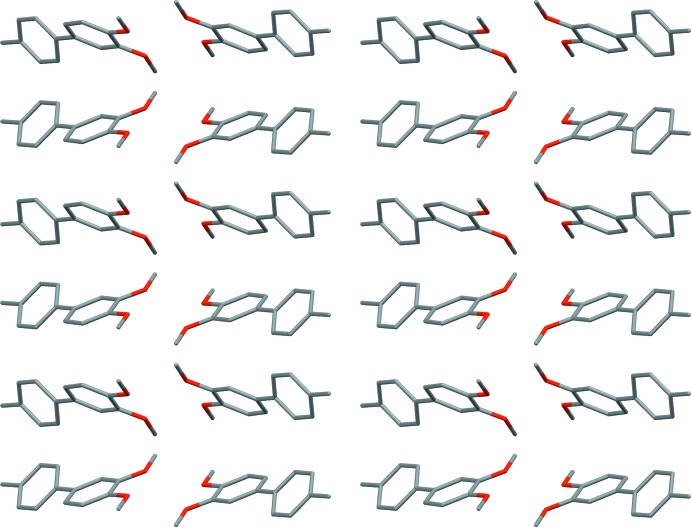
Packing of molecules along c axis. Hydrogen atoms are omitted for clarity.

**Fig. 5.**
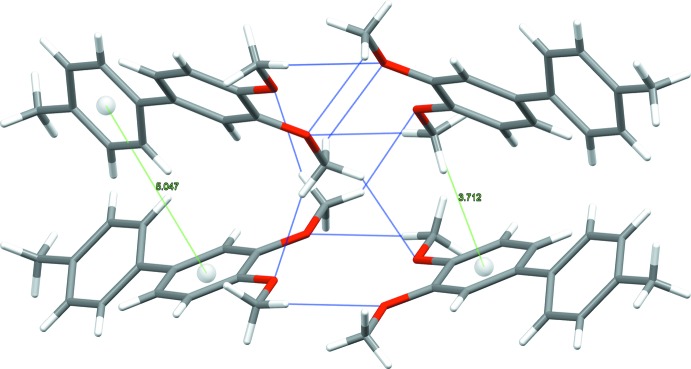
Weak hydrogen bond C–H···O (blue dashed lines), C–H···π and edge-to-face π–π contacts shown between the molecules.

## supplementary crystallographic information

### Comment

Percec-type biphenyl dendrons (Percec *et al.* 2006, 2007) are synthesized in a multi-step reaction sequence wherein the key synthetic step is the formation of *sp*^2^–*sp*^2^ carbon–carbon bonds. This is achieved using various cross-coupling reactions (Corbet & Mignani 2006). The title compound is synthesized in a gram-scale using the Suzuki-Miyaura cross-coupling reaction (Miyaura & Suzuki 1995), catalyzed by either palladium (Miyaura *et al.* 1981; Wolfe *et al.* 1999) or nickel (Percec *et al.* 2004, 2006). Biphenyl derivatives expand the scope and limitations of aryl ethers and esters (Nummelin *et al.* 2000) that serve as tectons for the construction of amphiphilic dendrons. Percec-type dendrons self-assemble into hollow and non-hollow cylindrical and spherical supramolecular dendrimers that further self-organize into hexagonal and cubic lattices (Percec *et al.* 2006, 2007, 2008; Peterca *et al.* 2006). As a contribution to a structural study of biphenyl derivatives (Lahtinen *et al.* 2013**a*,*b*,c*; Li *et al.* 2012**a*,b*) we report here the title compound 3,4-dimethoxy-4'-methyl biphenyl (I).

The compound (I) crystallizes in monoclinic *P*2_1_*/c* (No. 14) spacegroup having a single molecule in an asymmetric unit (Figure 1). The intramolecular dihedral angle between the phenyl rings is 30.5 (2)° [C(4)–C(5)–C(8)–C(9)] thereby being analogous to those found in similar biphenyls reported earlier (Lahtinen *et al.* 2013**a*,*b*,c*). The methoxy groups at 3- and 4-positions are co-planar in relation to the phenyl ring [C8>C13] with dihedral angles of -5.0 (2)° and 1.4 (2)°, respectively. Molecules are packed in head-to-head (methoxy-phenyl part) and tail-to-tail (methyl-phenyl part) formation (Figure 2) in zigzagged rows running parallel to (201) plane. By this, a columnar packing is formed in which methoxy and methyl layers alternate along *a*-axis (Figures 3 and 4). Network of C–H···π interactions occur between methoxy H atoms and phenyl groups with distance of 4.218 (2) Å. Infinite network of edge-to-face π–π interactions occur between phenyl rings (Figure 5) troughout the lattice along with (011) plane with distance of 5.047 (1) Å. Also weak C–H···O hydrogen bond network exist between the adjacent methoxy groups with D···A distances varying from 3.356 (2) to 3.694 (2) Å.

### Experimental

A dry Schlenk-tube was charged with 4-methylphenylboronic acid (6.0 g, 44.1 mmol), potassium fluoride (5.1 g, 88.3 mmol), 1-bromo-3,4-dimethoxybenzene (6.4 g, 29.4 mmol), Pd(OAc)_2_ (66 mg, 0.29 mmol, 1.0 mol%) and 2-(di-*tert*-butylphosphino)biphenyl (176 mg, 0.59 mmol, 2.0 mol%). The flask was sealed with a teflon screwcap, evacuated/backfilled with argon five times. Dry, degassed THF (40 ml) was added *via* syringe. The reaction mixture was stirred at ambient temperature until the aryl chloride had been completely consumed as judged by TLC analysis. The mixture was diluted with ether, filtered, and washed with 1*M* NaOH. The aqueous layer was extracted with ether, the combined organic layer was washed with brine and dried with Na_2_SO_4_. The crude product was purified by flash column chromatography: silica gel/CH_2_Cl_2_. Yield: 6.2 g (92%) of a white crystalline solid. Crystals suitable for a single-crystal structure determination were obtained from a slow evaporation of ethanol.

### Refinement

Hydrogen atoms were placed to their ideal positions as riding atoms (C host) using isotropic displacement parameters that were fixed to be 1.2 or 1.5 times larger than those of the attached non-hydrogen atom.

### Crystal data

**Table:**

C_15_H_16_O_2_	*F*(000) = 488
*M**_r_* = 228.28	*D*_x_ = 1.229 Mg m^−^^3^
Monoclinic, *P*2_1_/*c*	Cu *K*α radiation, λ = 1.5418 Å
*a* = 17.7430 (9) Å	Cell parameters from 1622 reflections
*b* = 8.7581 (3) Å	θ = 5.1–76.7°
*c* = 8.1135 (3) Å	µ = 0.64 mm^−^^1^
β = 101.795 (5)°	*T* = 123 K
*V* = 1234.17 (9) Å^3^	Plate, colourless
*Z* = 4	0.36 × 0.26 × 0.04 mm

### Data collection

**Table:**

Agilent SuperNova (Dual, Cu at zero, Atlas) diffractometer	2282 independent reflections
Radiation source: SuperNova (Cu) X-ray Source	1844 reflections with *I* > 2σ(*I*)
Mirror monochromator	*R*_int_ = 0.038
Detector resolution: 10.3953 pixels mm^-1^	θ_max_ = 69.0°, θ_min_ = 5.1°
ω scans	*h* = −18→21
Absorption correction: analytical (*CrysAlis PRO*; Agilent, 2010)	*k* = −10→7
*T*_min_ = 0.880, *T*_max_ = 0.977	*l* = −9→9
4273 measured reflections	

### Refinement

**Table:**

Refinement on *F*^2^	Primary atom site location: structure-invariant direct methods
Least-squares matrix: full	Secondary atom site location: difference Fourier map
*R*[*F*^2^ > 2σ(*F*^2^)] = 0.052	Hydrogen site location: inferred from neighbouring sites
*wR*(*F*^2^) = 0.143	H-atom parameters constrained
*S* = 1.07	*w* = 1/[σ^2^(*F*_o_^2^) + (0.0735*P*)^2^ + 0.0799*P*] where *P* = (*F*_o_^2^ + 2*F*_c_^2^)/3
2282 reflections	(Δ/σ)_max_ < 0.001
157 parameters	Δρ_max_ = 0.23 e Å^−^^3^
0 restraints	Δρ_min_ = −0.31 e Å^−^^3^
0 constraints	

### Special details

**Table:**

Geometry. All e.s.d.'s (except the e.s.d. in the dihedral angle between two l.s. planes) are estimated using the full covariance matrix. The cell e.s.d.'s are taken into account individually in the estimation of e.s.d.'s in distances, angles and torsion angles; correlations between e.s.d.'s in cell parameters are only used when they are defined by crystal symmetry. An approximate (isotropic) treatment of cell e.s.d.'s is used for estimating e.s.d.'s involving l.s. planes.
Refinement. Refinement of *F*^2^ against ALL reflections. The weighted *R*-factor *wR* and goodness of fit *S* are based on *F*^2^, conventional *R*-factors *R* are based on *F*, with *F* set to zero for negative *F*^2^. The threshold expression of *F*^2^ > σ(*F*^2^) is used only for calculating *R*-factors(gt) *etc*. and is not relevant to the choice of reflections for refinement. *R*-factors based on *F*^2^ are statistically about twice as large as those based on *F*, and *R*- factors based on ALL data will be even larger.

### Fractional atomic coordinates and isotropic or equivalent isotropic displacement parameters (Å^2^)

**Table:**

	*x*	*y*	*z*	*U*_iso_*/*U*_eq_	
O16	0.89457 (7)	0.46978 (13)	0.00350 (15)	0.0335 (3)	
O14	0.94276 (7)	0.60539 (14)	0.28726 (15)	0.0353 (3)	
C9	0.82177 (9)	0.56204 (18)	0.37788 (19)	0.0282 (3)	
H9	0.8400	0.6111	0.4829	0.034*	
C11	0.84258 (10)	0.47747 (17)	0.10680 (19)	0.0283 (4)	
C10	0.86951 (10)	0.55131 (17)	0.2626 (2)	0.0284 (3)	
C8	0.74672 (9)	0.50130 (17)	0.34160 (19)	0.0278 (3)	
C12	0.76904 (10)	0.41966 (19)	0.0695 (2)	0.0317 (4)	
H12	0.7506	0.3713	−0.0358	0.038*	
C13	0.72128 (10)	0.43187 (19)	0.1867 (2)	0.0310 (4)	
H13	0.6705	0.3918	0.1594	0.037*	
C4	0.70104 (10)	0.63163 (19)	0.58078 (19)	0.0312 (4)	
H4	0.7379	0.7101	0.5799	0.037*	
C3	0.65286 (10)	0.6385 (2)	0.6963 (2)	0.0344 (4)	
H3	0.6577	0.7212	0.7734	0.041*	
C6	0.64005 (10)	0.39913 (18)	0.4706 (2)	0.0317 (4)	
H6	0.6349	0.3161	0.3937	0.038*	
C7	0.59199 (10)	0.40748 (19)	0.5854 (2)	0.0340 (4)	
H7	0.5543	0.3303	0.5851	0.041*	
C5	0.69597 (9)	0.51097 (18)	0.46610 (19)	0.0281 (4)	
C2	0.59771 (10)	0.5262 (2)	0.7009 (2)	0.0331 (4)	
C1	0.54666 (11)	0.5331 (2)	0.8280 (2)	0.0418 (4)	
H1A	0.5696	0.6014	0.9203	0.063*	
H1B	0.5413	0.4305	0.8726	0.063*	
H1C	0.4958	0.5717	0.7737	0.063*	
C17	0.86955 (12)	0.3987 (2)	−0.1569 (2)	0.0401 (4)	
H17A	0.8250	0.4540	−0.2208	0.060*	
H17B	0.8551	0.2926	−0.1409	0.060*	
H17C	0.9114	0.4009	−0.2191	0.060*	
C15	0.97056 (11)	0.6901 (2)	0.4385 (2)	0.0429 (5)	
H15A	1.0228	0.7267	0.4391	0.064*	
H15B	0.9713	0.6241	0.5363	0.064*	
H15C	0.9366	0.7775	0.4435	0.064*	

### Atomic displacement parameters (Å^2^)

**Table:**

	*U*^11^	*U*^22^	*U*^33^	*U*^12^	*U*^13^	*U*^23^
O16	0.0440 (7)	0.0297 (6)	0.0324 (6)	−0.0001 (5)	0.0209 (5)	−0.0024 (4)
O14	0.0392 (7)	0.0340 (6)	0.0368 (6)	−0.0065 (5)	0.0173 (5)	−0.0074 (5)
C9	0.0380 (8)	0.0238 (7)	0.0255 (7)	0.0008 (6)	0.0128 (6)	0.0006 (5)
C11	0.0389 (8)	0.0230 (7)	0.0268 (7)	0.0044 (6)	0.0159 (6)	0.0036 (5)
C10	0.0362 (8)	0.0208 (7)	0.0307 (8)	0.0001 (6)	0.0129 (6)	0.0023 (6)
C8	0.0351 (8)	0.0235 (7)	0.0273 (7)	0.0017 (6)	0.0122 (6)	0.0024 (6)
C12	0.0406 (9)	0.0304 (8)	0.0256 (7)	0.0018 (6)	0.0101 (6)	−0.0017 (6)
C13	0.0351 (8)	0.0311 (8)	0.0285 (8)	−0.0003 (6)	0.0108 (6)	−0.0009 (6)
C4	0.0396 (9)	0.0286 (8)	0.0278 (7)	−0.0017 (6)	0.0124 (6)	−0.0003 (6)
C3	0.0448 (10)	0.0336 (9)	0.0278 (8)	0.0034 (7)	0.0146 (7)	−0.0020 (6)
C6	0.0391 (9)	0.0266 (8)	0.0318 (8)	0.0004 (6)	0.0131 (6)	−0.0003 (6)
C7	0.0360 (9)	0.0315 (8)	0.0382 (9)	0.0005 (6)	0.0166 (7)	0.0048 (6)
C5	0.0344 (8)	0.0263 (8)	0.0252 (7)	0.0030 (6)	0.0101 (6)	0.0036 (6)
C2	0.0365 (8)	0.0370 (9)	0.0285 (8)	0.0065 (7)	0.0130 (6)	0.0059 (6)
C1	0.0417 (10)	0.0537 (11)	0.0347 (9)	0.0078 (8)	0.0186 (7)	0.0044 (8)
C17	0.0522 (11)	0.0454 (10)	0.0264 (8)	0.0081 (8)	0.0165 (7)	−0.0011 (7)
C15	0.0435 (10)	0.0394 (9)	0.0497 (10)	−0.0106 (7)	0.0188 (8)	−0.0172 (8)

### Geometric parameters (Å, º)

**Table:**

O16—C11	1.3689 (19)	C3—H3	0.9500
O16—C17	1.429 (2)	C3—C2	1.393 (3)
O14—C10	1.359 (2)	C6—H6	0.9500
O14—C15	1.432 (2)	C6—C7	1.388 (2)
C9—H9	0.9500	C6—C5	1.400 (2)
C9—C10	1.388 (2)	C7—H7	0.9500
C9—C8	1.408 (2)	C7—C2	1.389 (3)
C11—C10	1.413 (2)	C2—C1	1.507 (2)
C11—C12	1.375 (3)	C1—H1A	0.9800
C8—C13	1.386 (2)	C1—H1B	0.9800
C8—C5	1.487 (2)	C1—H1C	0.9800
C12—H12	0.9500	C17—H17A	0.9800
C12—C13	1.401 (2)	C17—H17B	0.9800
C13—H13	0.9500	C17—H17C	0.9800
C4—H4	0.9500	C15—H15A	0.9800
C4—C3	1.393 (2)	C15—H15B	0.9800
C4—C5	1.399 (2)	C15—H15C	0.9800
			
C11—O16—C17	117.15 (14)	C5—C6—H6	119.5
C10—O14—C15	117.27 (13)	C6—C7—H7	119.3
C10—C9—H9	119.5	C6—C7—C2	121.46 (16)
C10—C9—C8	121.00 (15)	C2—C7—H7	119.3
C8—C9—H9	119.5	C4—C5—C8	121.97 (15)
O16—C11—C10	115.04 (14)	C4—C5—C6	117.45 (15)
O16—C11—C12	125.13 (15)	C6—C5—C8	120.57 (14)
C12—C11—C10	119.83 (14)	C3—C2—C1	120.94 (16)
O14—C10—C9	125.05 (15)	C7—C2—C3	117.72 (15)
O14—C10—C11	115.43 (14)	C7—C2—C1	121.33 (17)
C9—C10—C11	119.51 (15)	C2—C1—H1A	109.5
C9—C8—C5	120.98 (14)	C2—C1—H1B	109.5
C13—C8—C9	118.30 (14)	C2—C1—H1C	109.5
C13—C8—C5	120.72 (15)	H1A—C1—H1B	109.5
C11—C12—H12	120.0	H1A—C1—H1C	109.5
C11—C12—C13	120.10 (15)	H1B—C1—H1C	109.5
C13—C12—H12	120.0	O16—C17—H17A	109.5
C8—C13—C12	121.24 (15)	O16—C17—H17B	109.5
C8—C13—H13	119.4	O16—C17—H17C	109.5
C12—C13—H13	119.4	H17A—C17—H17B	109.5
C3—C4—H4	119.5	H17A—C17—H17C	109.5
C3—C4—C5	121.00 (16)	H17B—C17—H17C	109.5
C5—C4—H4	119.5	O14—C15—H15A	109.5
C4—C3—H3	119.4	O14—C15—H15B	109.5
C4—C3—C2	121.26 (15)	O14—C15—H15C	109.5
C2—C3—H3	119.4	H15A—C15—H15B	109.5
C7—C6—H6	119.5	H15A—C15—H15C	109.5
C7—C6—C5	121.10 (15)	H15B—C15—H15C	109.5
			
O16—C11—C10—O14	0.6 (2)	C4—C3—C2—C7	−0.4 (3)
O16—C11—C10—C9	−178.57 (13)	C4—C3—C2—C1	179.02 (16)
O16—C11—C12—C13	178.85 (14)	C3—C4—C5—C8	179.95 (14)
C9—C8—C13—C12	1.1 (2)	C3—C4—C5—C6	0.9 (2)
C9—C8—C5—C4	30.5 (2)	C6—C7—C2—C3	0.8 (3)
C9—C8—C5—C6	−150.44 (16)	C6—C7—C2—C1	−178.60 (16)
C11—C12—C13—C8	−0.2 (2)	C7—C6—C5—C8	−179.55 (15)
C10—C9—C8—C13	−0.7 (2)	C7—C6—C5—C4	−0.5 (2)
C10—C9—C8—C5	179.17 (14)	C5—C8—C13—C12	−178.85 (15)
C10—C11—C12—C13	−0.9 (2)	C5—C4—C3—C2	−0.4 (3)
C8—C9—C10—O14	−179.46 (15)	C5—C6—C7—C2	−0.4 (3)
C8—C9—C10—C11	−0.4 (2)	C17—O16—C11—C10	−178.82 (14)
C12—C11—C10—O14	−179.61 (14)	C17—O16—C11—C12	1.4 (2)
C12—C11—C10—C9	1.2 (2)	C15—O14—C10—C9	−5.0 (2)
C13—C8—C5—C4	−149.58 (16)	C15—O14—C10—C11	175.86 (15)
C13—C8—C5—C6	29.5 (2)		

### Hydrogen-bond geometry (Å, º)

Cg2 is the centroid of the C8–C13 ring.

**Table:**

*D*—H···*A*	*D*—H	H···*A*	*D*···*A*	*D*—H···*A*
C15—H15*A*···O16^i^	0.98	2.57	3.389 (2)	141
C15—H15*C*···O16^ii^	0.98	2.42	3.356 (2)	160
C4—H4···*Cg*2^ii^	0.95	2.96	3.7807 (18)	145
C17—H17*B*···*Cg*2^iii^	0.98	2.83	3.7119 (19)	150

Symmetry codes: (i) −*x*+2, *y*+1/2, −*z*+1/2; (ii) *x*, −*y*+3/2, *z*+1/2; (iii) *x*, −*y*+1/2, *z*−1/2.
